# A NineML-based domain-specific language for computational exploration of connectivity in the cerebellar granular layer

**DOI:** 10.1186/1471-2202-15-S1-P176

**Published:** 2014-07-21

**Authors:** Ivan Raikov, Shyam S Kumar, Benjamin Torben-Nielsen, Erik De Schutter

**Affiliations:** 1 Computational Neuroscience Unit, Okinawa Institute of Science and Technology, Onna-son, Okinawa, Japan; 2 University of Antwerp, Antwerp, Belgium

## 

The patterns of connectivity within a neuronal network can strongly influence its function. In neuroanatomical models of the cerebellum, the dimensions and topology of the neuronal arbors are crucial components as the cerebellum contains some of the most spatially extended cells in the brain (Golgi and Purkinje), which are connected by very long axonal projections of the granule cells (the parallel fibers). However, it is not known how these spatial features influence the overall cerebellar dynamics.

We begin to address this question by means of a prototype domain-specific language for constructing large-scale models of the cerebellar granular layer. The model currently implemented encompasses a patch of 1500 x 700 x 200 microns, and consists of 800 000 granule cells and 2000 Golgi cells. Input is provided by spatially embedded mossy fibers that function as non-homogeneous Poisson processes. The language framework is based on the emerging NineML network description language [1] and can interface to the NEURON simulator [2].

We show how the language can be used to investigate diverse neuroanatomical architectures. Variations of the basic granular layer architecture include random perturbations of the parallel fibers, exploring various shapes of synthetic Golgi cell morphologies and using experimentally obtained cell reconstructions. We characterize the effect of architecture changes on the network dynamics by comparing the spatial and temporal correlations of granule cell spiking activity.

The NineML language is intended to allow different methods for describing connectivity implemented as separate modules. Current working proposals include incorporating the Connection-Set Algebra [3], a general purpose graph library [1], and an equation-based format similar to the one implemented in the Brian2 simulator [4]. The present work demonstrates that an approach to describing connectivity based on geometric shape is compatible with the NineML object model.
